# Exploring mental health literacy among information technology (IT) professionals: Twitter content analysis

**DOI:** 10.1371/journal.pdig.0001078

**Published:** 2025-11-06

**Authors:** Edlin Garcia Colato, Yang Gao, Catherine M. Sherwood-Laughlin, Hongyi Zhu, Angela Chow, Sagar Samtani, Nianjun Liu, Jonathan T. Macy

**Affiliations:** 1 Department of Health and Wellness Design, Indiana University School of Public Health-Bloomington, Bloomington, Indiana, United States of America; 2 Department of Operations and Decision Technologies, Indiana University Kelley School of Business, Bloomington, Indiana, United States of America; 3 Department of Applied Health Science, Indiana University School of Public Health-Bloomington, Bloomington, Indiana, United States of America; 4 Alvarez College of Business, The University of Texas at San Antonio, San Antonio, Texas, United States of America; 5 Department of Epidemiology & Biostatistics, Indiana University School of Public Health-Bloomington, Bloomington, Indiana, United States of America; Regional Labor Institute, Directorate General Factory Advice Service and Labour Institutes (DGFASLI), Ministry of Labour and Employment, Government of India, INDIA

## Abstract

Mental health literacy has largely been studied via vignettes and surveys. Capturing the reality of the mental health literacy dimensions in a natural setting is an important step for moving towards a more actionable phase for mental health literacy. This study aims to identify the frequency patterns of the four mental health literacy dimensions reflected in the mental health-related tweets specific to information technology professionals. 15,782 tweets from October 2018 to October 2022 were collected from information technology-specific accounts. Content analysis, specifically a multi-class text classification approach, was used to analyze and interpret the tweets and categorize them into themes based on the mental health literacy construct. Tweets on “Knowledge and beliefs about risk factors and causes, self-treatments/interventions, and professional help available” were the most common (n = 6,179), and tweets on “ability to recognize specific disorders” (n = 196) were the least common. The ease of sharing content on X (formerly Twitter) could be leveraged to increase mental health awareness via targeted educational material on how to recognize specific disorders, seek help, and therefore improve mental health. Integrating mental health literacy information with the content being shared by well-established organizations in the information technology sector could help to enhance mental health literacy among information technology professionals.

## Author summary

Mental health literacy was first coined in 1997 by Anthony Jorm. The term includes four key areas that are a holistic understanding of mental health. We know, based on survey data, that levels of understanding about mental health vary among groups. However, we do not yet have a method for assessing mental health literacy in the natural world. Here, we took an interdisciplinary approach and used content analysis to test whether we could map tweets to the four dimensions of mental health literacy. We found most tweets were about understanding mental health risk factors and causes, self-treatment, and where to seek help. However, attitudes that promote recognition and appropriate help-seeking were represented by far fewer tweets. By working with well-known, trusted organizations, we can share helpful information to improve mental health knowledge among professionals. Our study provides new insights on which areas of mental health literacy (the ability to recognize specific disorders) need more direct focus when attempting to improve mental health literacy.

## Introduction

In 1997, Anthony Jorm, a professor of psychology in Australia, coined the term “mental health literacy” (MHL; [1]). MHL is derived from the larger umbrella term of health literacy, which is defined as finding, understanding, and using health-related information and its services [2]. The first definition for MHL was simply the “knowledge and beliefs about mental disorders which aid their recognition, management or prevention” [1]. The revised definition for MHL includes four major dimensions “the ability to recognise specific disorders; knowing how to seek mental health information; knowledge of risk factors and causes, of self-treatments, and of professional help available; and attitudes that promote recognition and appropriate help-seeking” [1]. MHL is the first step in the conceptual framework created by Jorm in achieving improved mental health [3].

### Mental health literacy assessments for adults

Since its inception, MHL has been assessed using vignettes that depict the symptomology of depression and schizophrenia as described in the International Classification of Diseases (ICD-10) and Diagnostic and Statistical Manual of Mental Disorders (DSM-IV) [1]. Vignettes were the primary instrument used for nearly two decades, becoming by default the prevailing and go-to assessment tool for MHL [4]. Vignettes are comprised of scenarios depicting a single person experiencing symptoms of a specific mental disorder, such as depression, anxiety, or schizophrenia [5]. Depending on the vignette response options, the respondent is asked to either state what condition is being depicted in the vignette in an open-ended manner or to select what they perceive to be the correct answer from a list of options [5]. After growing concern about the psychometric properties of the existing vignettes, new MHL measurement instruments were created [6–8]. MHL is now also assessed via scale-based assessment tools, such as the mental health literacy scale (MHLS) [7], the mental health literacy questionnaire regular [9], the short version for adults (MHLq-SVa) ages 18–25 [10], and expanded versions for healthcare professionals and students [11], as well as questionnaires specifically for university students [12,13]. All the scale-based measurement instruments require the respondent to recall from their memory and knowledge of the MHL information rather than from observation in real time. The natural world knowledge exchange can be derived from social network sites such as X (formerly Twitter). This is evident by previous research that has explored the extraction of such data and characterized it based on the nine attributes of Hodgkin’s Lymphoma [14]. Although the popularity of Twitter has been on the decline, the open sharing of text and immediate discussion following events is a key characteristic of other social network sites. Therefore, there is potential to translate these findings to other platforms. As of July 2023, Twitter was renamed to X; however, in this paper, we will continue to refer to the company as Twitter and its content as tweets because the data was collected before the rebranding of the company. To our knowledge, capturing real-world MHL (especially for particular industries, e.g., Information Technology) using social network sites has very limited exposition and has been constrained to investigating the causal relationship between MHL constructs using Twitter data [15].

Mental health conversations via social media platforms such as Twitter have been noted for their perceived therapeutic benefits [16]. The exchange of information and support is widespread on Twitter. Explicit focus on health literacy via the Twitter hashtag #healthliteracy was first identified on May 11, 2011 [17], and its increasing use highlights people’s interest in the topic. An exploratory study focused on depression and schizophrenia content posted on Twitter found that the majority of messages directed at depression were focused on resources or advertisements of services or products [18]. On the other hand, for schizophrenia, the content consisted of increasing awareness and dissemination of research [18].

### Purpose and research question

Capturing the reality of the MHL dimensions in a natural setting is an important step in moving towards a more actionable phase for MHL. To our knowledge, no study has focused on mapping social media content to the four major MHL dimensions to identify which are the most prominent in these public social media spaces, such as Twitter. This study used Twitter data known as tweets and was guided by the following research question: What are the frequency patterns of the four MHL dimensions as reflected in the mental health-related tweets specific to IT professionals?

This study was specifically designed to focus on Twitter within the IT professional contexts for two main reasons. First, Twitter was at the time a major social media venue for male professionals to express their feelings. Tweets, also known as microblogging, are particularly valuable amongst individuals who want to share information without engaging in direct conversation with specific people [19]. Second, MHL can easily be disseminated on social media platforms, just like other health promotion topics [20]. IT professionals are known to use Twitter to share information, making Twitter a viable source to explore their expression of MHL content. Furthermore, major social media platforms can reach large audiences, making them ideal for circulating MHL content and increasing efforts to improve MHL [21]. Reports of IT professionals experiencing burnout are plentiful [22–24], demonstrating the need for mental health-related resources and information, but MHL among the IT industry is understudied, especially in the U.S.

We hypothesized that the majority of the tweets would map to the recognition of mental disorders, knowledge of self-treatments, and attitudes that promote recognition and appropriate help-seeking because the tweets will be derived primarily from IT mental health advocacy groups and tweets focused on mental health, stress, anxiety, depression, and burnout. Past literature has found that at least for depression, the content shared on social media focuses on sharing resources that can help facilitate recognition and help-seeking [18]. However, only a few, if any, of the tweets will focus on knowledge of risk factors and causes and how to seek mental health information.

## Methods and materials

### Qualitative approach and research paradigm

The goal of this study was to analyze and interpret the Twitter text content, hereinafter referred to as “tweets,” and categorize it into themes based on the MHL construct. To accomplish this goal, content analysis was selected as the qualitative approach for this study. The associated research paradigm is interpretivism because we sought to understand how IT Twitter users express their understanding of mental health as it relates to the four dimensions of the MHL construct.

#### Context.

**Fig 1:**
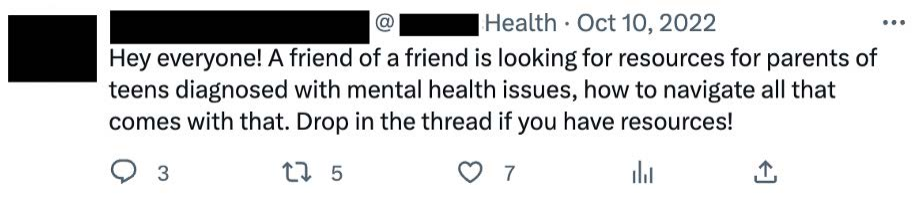
Screenshot example of tweet showing a dimension of MHL content.

Founded in 2006, Twitter, now known as X, is a social media platform with more than 220 million active users [25,26]. Twitter was selected as the social media platform of interest because, at the time of data collection, it had previously been noted as a space individuals feel comfortable using to exchange mental health discussions [16]. The population of interest, IT professionals, extensively use Twitter to share content, resources, and materials for other professionals to consider. Increasingly, there has been a greater usage of Twitter from IT professionals reporting cyber-attacks (e.g., early warnings), sharing appropriate resources, and requesting mental health support for themselves (either directly or through friends). An example of such a posting is presented in Fig 1.

#### Sampling strategy.

Tweets from October 2018 through October 2022 were selected based on the origin date of a known account focused on mental health awareness for IT professionals. The end date of October 2022 was selected to provide coverage of content spanning a total of four years that included time before, during, and after the peak of the COVID-19 pandemic. The criteria for deciding when no further sampling was necessary were based on the data availability that matched the keyword search for the selected data range, which is further elaborated in the data collection methods section below.

#### Ethical issues pertaining to human subjects.

Though API access has become restricted in recent years, Tweets remain publicly available data. While informed consent is not required for public data, all data are treated with the same ethical care as if it were private. To qualify as an exempt study as specified by Indiana University’s (IU’s) Human Research Protection Program (IRB), usernames and personally identifiable information (PII) of the individuals and groups who posted the tweets were redacted. Due to the sensitive nature of this topic being about mental health, the research team took extra care in ensuring that even names, usernames or other PII mentioned within tweets were redacted for added confidentiality. This study (Protocol #18254) was approved as an exempt study by IU’s IRB. Results were reported in aggregate form only.

### Data collection methods

Twitter data, specifically tweets and replies to tweets, were collected via Twitter’s Application Program Interface (API) for October 2018 – October 2022. The API used for collection was an approved academic-level developer access (and was accessible at the time of executing the initial analysis). We executed data collection using two strategies for the proposed content analysis: account-based search and keyword-based search. For the account-based search, we consulted IT professionals with over three decades of experience in the industry to identify three mental health awareness accounts geared towards IT professionals. However, at the time of the data collection in early 2023, two identified accounts had been either removed or de-activated. The remaining account contained 1,358 tweets. In our second strategy, we sought to leverage a keyword-based approach to capture a wider view of general mental health discussion among IT professionals. In particular, we input several keyword combinations into the Twitter API to retrieve tweets. The searches included the following keyword combinations: [IT professional OR IT industry OR cybersecurity OR cyber security] AND [anxiety OR depression OR depressed OR mental health OR burnout] for the October 2018-October 2022 date range. In total, the data collection contains n = 15,782 public tweets and re-tweets posted within the four-year period.

#### Units of study.

Tweets were retrieved with pre-selected meta-data based on the following fields (Table 1): id, created_at, language, referenced_tweets, retweet_count, reply_count, like_count, quote_count. Although no Twitter username information was retained for the data analyzed, the identification field was retained as it is comprised of a unique identifier of the requested tweet to help retrieve a specific tweet. Having this unique identifier also assisted in identifying a tweet that may have been retrieved more than once based on the keyword choice. This allowed for duplicates to be removed. To map out the distribution of tweets by time, the field “created_at” (i.e., tweet creation timestamp) was retained. The analysis was limited to English-only tweets to control the scope and help facilitate better interpretability of the results.

**Table 1:** Twitter data field type, description, and possible usage.

Field	Description	How it can be used
id	Unique identifier of requested tweet	Used to retrieve a specific tweet
created_at	Creation time of tweet	Time-series analysis
language	Language of the tweet	Filter the relevant languages
referenced_tweets	A list of tweets a tweet refers to	Used to understand conversational aspects of retweets etc.
retweet_count	Public engagement metrics for the tweet at the time of the request	Used to measure tweet engagement
reply_count		
like_count		
quote_count		

#### Data pre-processing and analysis.

A multi-class text classification approach was selected to automatically categorize each tweet exclusively into a MHL category based on its content. Multi-class text classification is a well-established method in machine learning and deep learning literature and has been used extensively in mental health applications [27]. Compared to this method, manual content analysis, which involves assigning each tweet to an MHL category manually, is less efficient for handling large volumes of tweets, being both time-consuming and prone to errors. Multi-class text classification operates with several steps. First, a gold-standard dataset is developed that captures and represents the different classes that a data point (e.g., tweet) could be categorized into. Second, features or embeddings (i.e., vector representations) are extracted from each data point. Third, the features or embeddings (in this case the tweets) are inputted into machine learning or deep learning models that are trained using strategies such as ten-fold cross-validation or hold-out sampling on the developed gold-standard dataset. Fourth, model performances are evaluated based on well-established metrics such as accuracy, precision, recall, and/or F1-score. Finally, the model with the best performance is applied (i.e., inference stage) onto unseen data to predict or categorize each data point into one of the pre-defined categories.

To facilitate the proposed multi-class text classification analysis, we needed to first develop a gold-standard dataset to train each machine learning or deep learning classifier. Therefore, we developed a gold-standard dataset with 790 tweets across four dimensions of the MHL construct. Data were first independently labeled by two certified Mental Health First Aiders (MHFA) with over a decade of mental health experience using a pre-established codebook with the following labels: 0 = The ability to recognize specific disorders (n = 46); 1 = Attitudes that promote recognition and appropriate help-seeking (n = 104); 2 = Knowledge and beliefs about risk factors and causes, self-treatments/interventions, and professional help available (n = 98); 3 = Knowledge of how to seek mental health information (n = 55), and; 4 = Not relevant (n = 487). An example of a not relevant tweet is one that included the hashtag #depression but the tweet was that of an image or other multimedia that was not text and therefore could not be analyzed for the content’s relevancy to MHL. Given the length of tweets (240 characters), we assigned each tweet into one category (i.e., MHL dimension) only. The pre-determined threshold of acceptability was set at 0.80-1 which represents an almost perfect reliability [28,29]. Authors 1 and 4 met to discuss and resolve discrepancies in labels (n = 71) having an initial 91% agreement. Differences in coding were found because of certain tweets having content covering more than one MHL dimension. Author 6, MHFA certified, reviewed the final codes for agreement. Samples of labeled tweets by MHL construct are presented in Table 2.

**Table 2:** Label, codes, and sample tweets by label type.

Label	Illustrative tweet example (Paraphrased to maintain anonymity)
0 = The ability to recognize specific disorders	Do you know how to spot the signs of anxiety, stress or depression? Join us next week as we discuss practical ways in which to help yourself & others spot & deal with these feelings.
1 = Attitudes that promote recognition and appropriate help-seeking	Recognizing depression is a good first step. Let us know if you want to talk or point you to some professional info.
2 = Knowledge and beliefs about risk factors and causes, self-treatments/interventions, and professional help available	Bikes will lower your risk of things like depression, obesity, or having a heart attack.
3 = Knowledge of how to seek mental health information	We’re reaching out to the professional behavioral health contacts we have for more information.
4 = Not relevant	“*does a dance*”

Following gold-standard dataset construction, we pre-processed all collected tweets to help normalize any inconsistencies and remove erroneous content. Our pre-processing pipeline included tokenizing tweets based on their white space (e.g., “mental health” would become two tokens, “mental” and “health”), lowercasing tokens, stemming tokens to their root form (e.g., “computer” to “compute”), and removing stop words that add little semantics to the content of a tweet (e.g., “a,” “that,” “the,” etc.). Pre-processing tweets in this fashion for multi-class text classification is a commonly accepted practice in social media analytics literature [27].

Model training requires extracting features or embeddings from each tweet. Rather than employing manual feature engineering efforts (often time consuming, ad-hoc, and/or error-prone), we generated embeddings using Bidirectional Encoder Representations from Transformers (BERT)-based embeddings for each tweet to vectorize the tweets [30]. BERT is a state-of-the-art approach for extracting embeddings from text data [30]. The embeddings were then inputted into the text classification models. To ensure a thorough overview of model performances, we adopted two major categories of text classification models commonly used in past mental health literature [27]: classical machine learning and deep learning. The classical machine learning model’s category included four models: Logistic Regression (LR), Random Forest (RF), kernel-Nearest Neighbors (KNN), Support Vector Machine (SVM). Our deep learning model category included four models: Long Short-Term Memory (LSTM), Bidirectional Long Short-Term Memory (BiLSTM), Gated Recurrent Unit (GRU), and Convolutional Neural Network (CNN).

For the training procedure, 10-fold cross validation was used. The performance metrics include accuracy, precision, recall, and F1-score. Each metric uses a combination of True Positive (TP), False Positive (FP), True Negative (TN), and/or False Negative (FN) rates in their formulations. The specific formulas for these computations appear below [31]:

$Accuracy=\frac{TP+TN}{TP+TN+FP+FN},\;Precision=\frac{TP\;}{TP+FP\;}\backslash )$


$$Recall=\frac{TP}{TP+FN},\;F1-score=\frac{2\times Precision\times Recall}{Precision+Recall}\mathrm{\backslash ]}$$

Each metric produces a scalar value between 0 and 1. Higher performance values indicate stronger performance. Since the gold-standard dataset is imbalanced (i.e., significantly more instances of data in one category compared to others), F1-score is the best metric to evaluate overall performance [32]. We also computed the Area Under the Receiver Operating Characteristic Curve (AUROC) as an additional metric that calculates the integral of the ROC curve. Given the range of hyperparameters that are often available for tuning machine learning models, we used a grid search approach to identify the model configurations that attained the best performance. All implementations were executed using the Python programming language. Classical machine learning models were implemented using the scikit-learn package [33]. The deep learning models were implemented using Keras [34]. All training and testing processes were conducted on IU’s Big Red 200 supercomputer [35].

## Results

In this section, we present two sets of results. The first set of results reports the performances of the trained classifiers. In the second set of results, we report the results attained by applying the trained classifiers on the larger collection of tweets.

### Classifier performances

In this sub-section, we present the performances for each classifier. In Table 3, we report each metric by model type (classical machine learning vs deep learning). The best performances are indicated by bold face font.

**Table 3:** Classification models and corresponding results for performance metrics.

Model Type	Model	Accuracy	Precision	Recall	F1-Score	AUROC
Classical Machine Learning	Logistic Regression	72.15%	68.70%	72.15%	68.95%	**92.89%**
	K-Nearest Neighbors	67.72%	64.55%	67.72%	65.59%	81.20%
	SVM	73.41%	70.29%	73.42%	71.10%	92.79%
	Random forest	65.82%	63.80%	65.82%	62.79%	84.45%
Deep Learning	GRU	65.82%	59.88%	65.82%	61.62%	87.33%
	CNN	73.42%	69.42%	73.42%	70.22%	91.61%
	LSTM	**75.95%**	**75.87%**	**75.95%**	**73.55%**	91.89%
	BiLSTM	72.15%	69.93%	72.15%	69.75%	91.81%

Note. All scores are based on the test dataset, and can also be reported as Test Accuracy, Test Precision, Test Recall, and Test F1-Score.

Overall, the classical machine learning methods attained F1-scores between 62.79% and 71.10%. SVM was the best performing classical machine learning model with a 71.10% F1-score. Deep learning models showed similar variability, with models producing F1-scores between 61.62% and 73.55%. The best performing deep learning model in terms of F1-score was LSTM (73.55%). This performance was slightly higher than SVM’s performance. This performance could be attributed to several factors, including the length of tweets (which tend to be shorter than other health documents, e.g., electronic health records) as well as the size and imbalanced nature of the gold-standard datasets, and tweets with content that spans multiple MHL dimensions. Future research can examine multi-label classification (i.e., having a single tweet assigned to multiple categories). Additional research that examines more advanced machine learning paradigms (e.g., knowledge distillation, transfer learning) could be promising to help boost model performance.

#### Categorized tweets.

Following model training and testing, we applied the classifiers onto the entire set of collected tweets to assign a label to each tweet. Conventionally, the best-performing classifier is applied to the larger corpus of data to assign labels (i.e., perform inference). In this study on our dataset, LSTM was the best performing classifier (consistently across tracked performance metrics) and was therefore used for inference. The overall categorization of the results is presented in Table 4. The count of tweets that were in our gold-standard dataset were not included in the overall count reported in the table.

**Table 4:** Tweet counts for each MHL construct label and the not relevant (n = 14,992).

Label Code	Label	Count
0	The ability to recognize specific disorders	196
1	Attitudes that promote recognition and appropriate help-seeking	1,111
2	Knowledge and beliefs about risk factors and causes, self-treatments/interventions, and professional help available	6,179
3	Knowledge of how to seek mental health information	4,131
4	Not Relevant	1,375

Overall, the MHL dimension of “knowledge and beliefs about risk factors and causes, self-treatments/interventions, and professional help available” had the most tweets (n = 6,179) out of the total relevant tweets (n = 13,617). This suggests that many of the tweets indicated that the IT professional community has knowledge and/or beliefs about the types of jobs and activities that cause mental health concerns such as depression or anxiety. The category of “knowledge of how to seek mental health information” had the second most tweets. This suggests that the IT professional community has some knowledge about how to identify possible resources to support individuals who may have mental health concerns. Similarly, this also indicates that IT professionals are actively seeking materials to support their mental health. The categories of “the ability to recognize specific disorders” and “attitudes that promote recognition and appropriate help-seeking” had the least (n = 196) and second least number of tweets (n = 1,111), respectively. This suggests that the IT professional community may have difficulty in identifying mental health disorders within themselves or others via this public forum.

**Fig 2:**
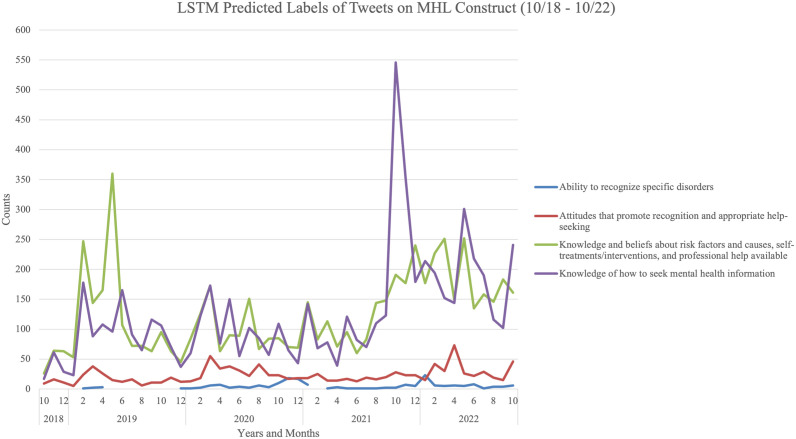
Predicted Labels of Tweets on MHL Construct (10/18 - 10/22).

In addition to reporting the classified tweets at an aggregate level, we sought to organize the classified results based on the metadata we collected in a manner that could have practical utility for the IT professional community. While one ideal way of doing this is to organize classified tweets based on the poster of the tweet, we could not include the names of any posters in our analysis. Thus, we sought to present the tweets in an alternative fashion, namely, in a temporal manner. This particular presentation could have unique value for IT professionals for several reasons. First, IT professionals work in a real time, “around the clock” fashion. Twitter is unique in that tweets are posted at a much higher velocity than posts in other social media platforms [36]. Second, IT professionals consume knowledge about critical information such as cyber-attacks that are often reported on Twitter, given the platform’s strengths. Finally, and relatedly, knowledge about how to tackle the issues (including how to address some of the mental health concerns that may arise) that often stem from cyberattacks is often shared via Twitter after the event. For these reasons, we present the results of the classified tweets over time in Fig 2.

We visualized the results on a monthly basis from October 2018 to October 2022. These results demonstrate that content focused on the “recognition of specific disorders” and “attitudes that promote recognition and appropriate help-seeking” are consistently the least common type of MHL content among the four constructs being publicly discussed by IT professionals on Twitter. On the other hand, “knowledge and beliefs about self-risk factors and causes, treatments/self-help interventions, professionals help available” and “knowledge of how to seek mental health information” are the two most commonly discussed and appear to not only have spikes throughout the entire selected date range, but especially in late 2021.

There was an increase (aka “spikes”) in tweets across all four categories in February 2019, January 2020, May 2020, January 2021, October 2021, and May 2022. The biggest spike for “knowledge and beliefs about self-risk factors and causes, treatments/self-help interventions, professionals help available”, the category with the most tweets, was in May 2019. Tweets during that time were circulating on how “increasing pressure, workload and budgetary deficits, leading to a quarter of #CISOs worldwide suffering from physical or mental health issues.” On the other hand, the largest spike overall was for the category “knowledge of how to seek mental health information” in October 2021.

## Discussion and conclusion

### Principle findings and implications

Our study categorized Twitter content about mental health and IT professionals and assessed trends of content as it relates to the four major dimensions of the MHL construct. We used classical machine learning and deep learning tools to categorize four years-worth of collected tweets in the four well-established dimensions of the MHL construct. Our findings highlight that the content about mental health, depression, anxiety, and burnout among IT professionals has consistently focused more on two areas of MHL (1) knowledge and beliefs about risk factors and causes, self-treatments/interventions, and professional help available and 2) knowledge of how to seek mental health information) and less on the other two (3) ability to recognize specific disorders and 4) attitudes that promote recognition and appropriate help-seeking) MHL dimensions throughout all four years (October 2018-October 2022).

Interestingly, there are some time periods (e.g., February 2019, January 2020, October 2021, May 2022) in which there was an increase in tweets representing all four categories. Further investigation of the shared times of spikes in February 2019 revealed that the content being frequently shared and re-shared by IT professionals on Twitter include cybersecurity burnout, how to recognize the signs and symptoms, how to reduce the risk, where to seek help, and how to seek information. In particular articles describing burnout, depression, and anxiety and warnings of the risks, describing how to identify the symptoms and seek help were being re-circulated throughout the month of February 2019 [37].

In October 2021, a large overall spike for the category “knowledge of how to seek mental health information” was found. This result is not surprising because October is the cybersecurity awareness month, and October 10^th^ is World Mental Health Day. In October 2021, there were two major industry articles published within a week of each other covering mental health in the cybersecurity space. Cisco’s article titled “Mental Health & Burnout in Cybersecurity: Tips, Stories and Insights” was published on October 6, 2021, and on October 19, 2021, DarkReading published an article titled “The Simmering Cybersecurity Risk of Employee Burnout” – accounting for more than 300 of the 441 posted tweets and re-tweets for the month of October 2021.

In the multi-class text classification analysis of the mental health and IT professional tweets, the theme of the type of content most readily shared emphasizes that the IT professional community appears to be keen on receiving information about mental health that is provided from trusted sources such as Cisco and DarkReading. Based on the re-tweets, it appears that IT professionals are also willing to re-share the “mental health and IT professional” content with their own followers. In particular, the type of content being shared is predominantly about burnout compared to the risks for depression and anxiety. Considering the trust IT professionals have towards major professional organizations might put more of the impetus on these well-regarded sources to produce valid and reliable MHL content geared at the IT community. Furthermore, since most of the collected mental health content being shared amongst IT professionals, at least for this four-year time period, is about “knowledge and beliefs about risk factors and causes, self-treatments/interventions, and professional help available”, this suggests that online communities might be a viable option to share this type of information going forward. This is especially true because IT professionals are not only re-tweeting content from these sources but are also willing to express their own thoughts and comments about the published information about risk factors, causes, self-treatments, and interventions.

Previous mental health research using Twitter data has largely focused on identifying the mental illness being described via the tweets [38]. This study, however, takes a closer look at what aspects of MHL are being discussed online, especially when it pertains to the most prevalent mental health conditions (anxiety, depression, and burnout) that are of concern for working IT professionals. By knowing what areas of the MHL are least covered via this public discourse channel, there might be an opportunity for the mental health awareness accounts on Twitter to increase targeted coverage of all aspects of MHL.

### Limitations

Some limitations must be noted. First, the data collected did not include usernames making us unable to decipher how many of the tweets were posted from the same account. By not having usernames, we were also unable to identify how the tweets were related to each other based on which accounts were retweeting from the original tweet and each other. However, the selected topic of mental health meant that there could have been some sensitive information collected from the tweets. The sensitivity of information made it essential to redact any identifiable information to safeguard users who posted the tweets, even if the content was publicly available. This ensured anonymity when someone was describing a condition, whether it was their own signs and symptoms or those of a known person, for example. Second, although all tweets gathered during the four-year period met the specified account type and/or keyword search criteria and have been mapped to the MHL dimensions, the content itself was not assessed for accuracy or reliability. In other words, there may be content being shared about risk factors and causes that is misinformation that would require correction via more actionable MHL materials. As Twitter is a public platform, people interested in entering the IT industry will have access to this content, which might affect their perception of the risk factors. The MHL category, knowledge and beliefs about risk factors and causes, self-treatment/interventions, and professional help available, for instance, do not distinguish whether knowledge and beliefs are based on fact. This MHL category in particular would be most prone for misinformation and would benefit from further exploration to identify potential biases in personal accounts. Lastly, tweets can sometimes cover more than one dimension of MHL which is not captured by the prediction models. Rather, the tweet is grouped based on which dimension is most relevant to the majority of information found in the tweet. Future studies could further explore multi-label multi-classification methods.

### Public health implications

Leveraging the ease of sharing content on Twitter to increase mental health awareness and provide more targeted educational material on how to recognize specific disorders could guide people towards relevant help-seeking resources and therefore help to improve mental health. Especially as social media continues to be leveraged as a strategy to disseminate health information [39]. In an environment where there still remains stigma around the topic of mental health [40], identifying ways to share MHL content to a larger audience is crucial in the goal of increasing the number of individuals who take action and seek the services they need. A more accepted manner of obtaining MHL could be accomplished by integrating MHL information with the content being shared by well-established organizations in the IT industry.

These findings can be used to inform targeted MHL campaigns and training produced for organizations. For instance, the emerging theme of there being less content shared about abilities of recognizing mental disorders compared to the other four dimensions is an opportunity to provide training on identifying early warning signs in an ethical and respectful manner. As such the impact of increased awareness and self-identification of symptoms could translate into reduced stigma, increased early help-seeking, and improvement among employee retention and morale.
